# Cerium Dioxide Nanoparticles Modulate Ribosomal DNA Transcriptional Activity in Cultured Human Skin Fibroblasts

**DOI:** 10.3390/ijms27198913

**Published:** 2026-10-07

**Authors:** Natalia N. Veiko, Elizaveta S. Ershova, Madina M. Sozarukova, Elena V. Proskurnina, Larisa V. Kameneva, Edmund V. Kostyuk, Tatiana M. Rozhnova, Yuriy L. Orlov, Svetlana Yu. Vikhrova, Alesya M. Drozhdinina, Vladimir K. Ivanov, Svetlana V. Kostyuk

**Affiliations:** 1 Petrovsky Russian Scientific Center of Surgery, Institute of Biology of Aging and Healthy Longevity Medicine with Clinic of Predictive Medicine, Moscow 119435, Russia; satelit32006@yandex.ru (N.N.V.); es-ershova@rambler.ru (E.S.E.); kamlar@med-gen.ru (L.V.K.); svet-vk@yandex.ru (S.V.K.); 2 Kurnakov Institute of General and Inorganic Chemistry of the Russian Academy of Science, Moscow 119435, Russia; s_madinam@bk.ru (M.M.S.); proskurnina@gmail.com (E.V.P.); edmundvk@ya.ru (E.V.K.); alesya0400@mail.ru (A.M.D.); van@igic.ras.ru (V.K.I.); 3 Center of Digital Health and AI in Medicine, I.M. Sechenov First Moscow State Medical University of the Ministry of Health of the Russian Federation ( Sechenov University), Moscow 119991, Russia; stm-i@yandex.ru

**Keywords:** ribosomal DNA, rDNA transcription, cerium dioxide nanoparticles, gene expression, human skin fibroblasts

## Abstract

The study investigates the effect of cerium dioxide (CeO_2_) nanoparticles on ribosomal DNA (rDNA) transcriptional activity and the status of ribosomal genes in human skin fibroblasts. Nano-CeO_2_ has attracted attention due to its antioxidant and redox properties; however, its impact on rDNA has so far remained virtually unexplored. Fibroblasts from ten adult donors were incubated with nano-CeO_2_ for 3 and 24 h, and the copy number of ribosomal genes and their functional state were assessed using real-time quantitative PCR and non-radioactive quantitative hybridization; the interaction of the nanoparticles with the cells was also assessed by fluorescence microscopy. It was shown that exposure to CeO_2_ nanoparticles leads to functional changes in rDNA transcription and rRNA abundance, indicating modulation of ribosomal biogenesis and consistent with a model of altered balance between transcriptionally active and inactive rDNA copies, although this study did not directly measure ribosome content or rDNA fraction distribution. The observed effects are likely mediated by the antioxidant and redox properties of the nanoparticles. Thus, nano-CeO_2_ can be considered a compound capable of modulating the functional state of ribosomal genes in normal human cells.

## 1. Introduction

Cerium oxide nanoparticles (nano-CeO_2_) have pharmacological potential because of their antioxidant properties [1]. Redox cycling between Ce(3+) and Ce(4+) allows nano-CeO_2_ to catalytically react with oxygen-containing radicals, mimicking the behavior of antioxidant enzymes such as superoxide dismutase and catalase [2,3]. Many authors believe that nano-CeO_2_ may combat chronic inflammation and pathologies associated with chronic oxidative stress, including cancer and neurodegeneration [4,5,6]. The use of primary human skin fibroblasts as a cellular model in this study is justified by several considerations. First, the skin represents the primary barrier for dermal and systemic exposure to nanoparticles, and dermal fibroblasts are a key cell population responding to nanoparticle application, particularly in the context of sunscreen formulations, wound dressings, and transdermal therapeutic systems. Second, skin fibroblasts are widely employed as a model for studying oxidative stress, cellular senescence, and photoaging, and nanoceria have already demonstrated photoprotective effects in this cell type [7]. Third, cerium oxide nanoparticles are being explored as potential therapeutic agents for age-associated skin disorders and chronic inflammatory conditions, where fibroblast dysfunction plays a central role. Finally, because ribosomal biogenesis in skin fibroblasts is known to be altered during aging and stress responses, this model provides a relevant system to investigate the impact of nanoceria on key homeostatic mechanisms with direct implications for geroprotective strategies. Numerous studies have examined the response of human cells to nanoceria [8]. The antioxidant and redox properties of CeO_2_ nanoparticles have attracted considerable interest for biomedical applications, including photoprotection of skin fibroblasts [7], protection against oxidative stress-induced damage [9], and potential therapeutic use in conditions associated with chronic inflammation and oxidative stress [4]. However, the biosafety profile of nanoceria remains a subject of ongoing debate. While several studies have reported low cytotoxicity and favorable biocompatibility in various cell types [4,10], other investigations have demonstrated toxic effects in different organ systems. In vivo studies have shown that cerium oxide nanoparticles can accumulate in the liver, spleen, and kidneys, inducing oxidative stress, inflammation, and apoptosis in these organs [11,12]. Pulmonary exposure to CeO_2_ nanoparticles has been associated with acute oxidative stress, DNA damage, and inflammatory responses [11]. Furthermore, systematic reviews have highlighted unresolved questions regarding the biosafety and potential long-term toxicological mechanisms of nanoceria, emphasizing that surface modifications, particle size, and exposure routes significantly influence their biological effects [13,14]. Thus, while CeO_2_ nanoparticles hold promise for therapeutic applications, a comprehensive assessment of their safety profile, particularly in the context of ribosomal function and nucleolar activity, remains essential. There are reports devoted to the effects of nanosized CeO_2_ on cellular energy structures, namely mitochondria [15], but there are almost no studies addressing the influence of nano-CeO_2_ on another important process in eukaryotic cells, ribosome biogenesis, which is an integral part of the protein synthesis system.

**Figure 1:**
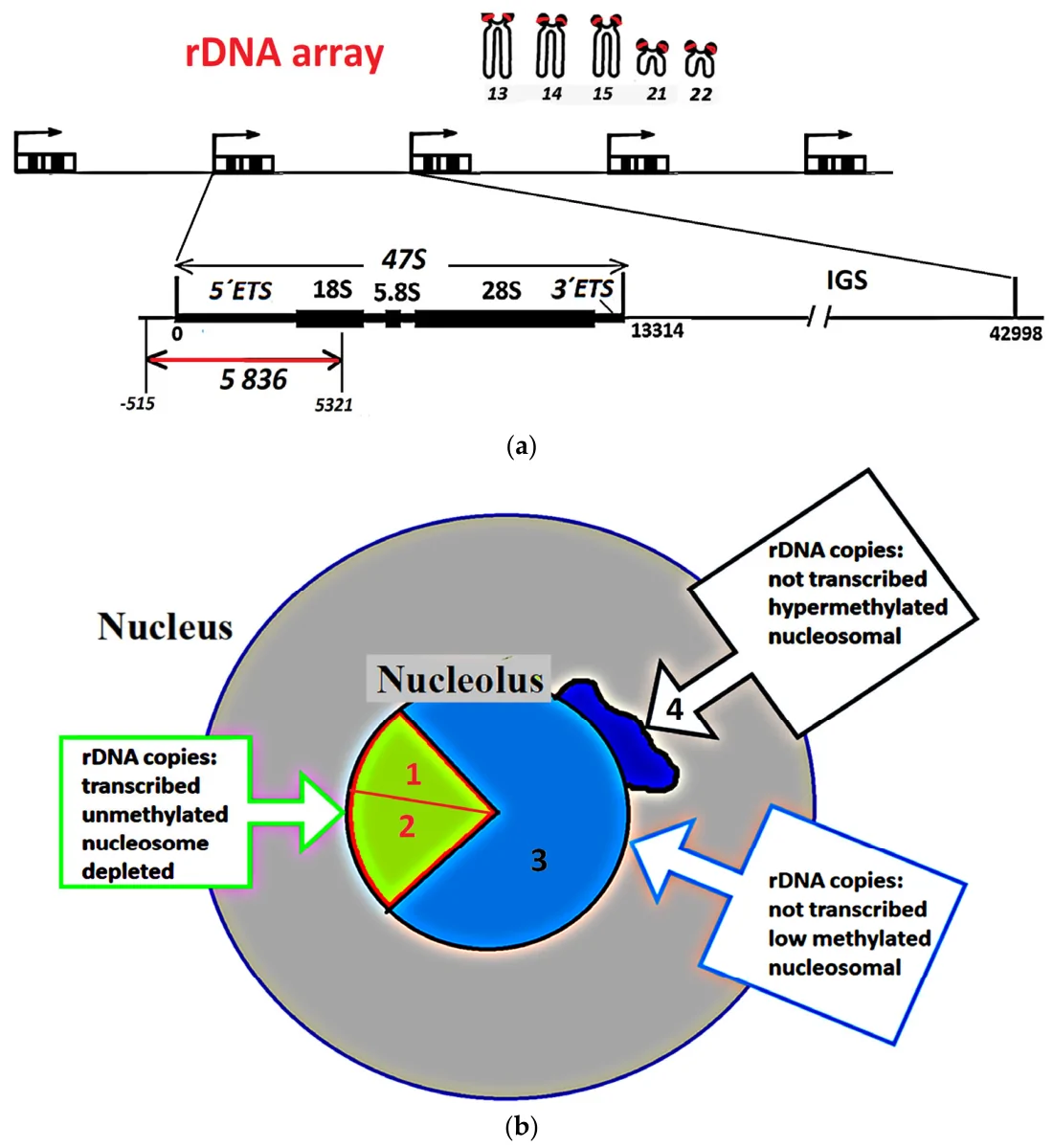
(**a**) Scheme of the ribosomal repeat; the region detected by the DNA probe is indicated. (**b**) Fractions of ribosomal genes within the nucleolus.

To study nanoparticles targets we consider ribosomal genes. The human ribosome consists of two components: ribosomal RNA (rRNA) and approximately 70–80 ribosomal proteins [16]. The genes encoding 28S, 5.8S, and 18S rRNA (rDNA) in the human genome are represented by multiple tandem repeats. On average, ~400 rDNA copies are organized in tandem repeats of 43 kb on five pairs of acrocentric chromosomes. Each repeat unit includes a transcribed region containing the 28S, 5.8S, and 18S rRNA genes (47S rRNA) spanning 13.3 kb and a non-transcribed intergenic spacer (Figure 1). Together with 5S rRNA copies (whose genes are located on chromosome 1), these rRNAs form ribosomes [17]. The main function of ribosomal repeats is to synthesize rRNA for ribosomes. rDNA transcription is carried out by RNA polymerase I in a special nuclear structure, the nucleolus. rDNA copies can be divided into four fractions [18,19], shown in Figure 1b. Fractions 1 and 2 are referred to as potentially active rDNA copies. These copies are unmethylated and do not contain nucleosomes. The size of fraction 1 (the copies actually transcribed at a given time) depends on the cell’s need for ribosomes. During active cell growth, this fraction increases. Roughly one third of mammalian rDNA copies are potentially active [18,20]. Fraction 3 comprises inactive rDNA copies. These copies are located in the same nucleolar structures as the potentially active copies and are required for normal nucleolar function.

Copies in fraction 3 are inactive because of methylation of certain CpG sites in the rDNA promoter region [17]. These copies contain nucleosomes. Fraction 4 includes inactive hypermethylated rDNA copies. The entire transcribed rDNA region, not only the promoter, is methylated. These copies contain multiple mutations, are localized at the nucleolar periphery, and are part of the heterochromatin surrounding the nucleolus. The maximal amount of rRNA that a cell can synthesize in response to various stimuli depends on the number of potentially active copies (fractions 1 and 2 combined). In turn, the number of potentially active copies depends on the total number of rDNA copies in the cell.

The human genome contains from ~200 to ~1000 copies of ribosomal genes [21,22,23]. Data on the stability of rDNA content in human and animal cells under stress are quite contradictory. Some authors have reported that the total number of rDNA copies in tissues and cultured cells can change under the action of organic compounds and metal salts that affect redox processes [24,25,26,27]. These changes are directly related to the ability of cells to respond to stress by increasing ribosome abundance. Other authors, including the authors of the present study, postulate the stability of rDNA content in the genome under oxidative stress caused by pathology or by damaging agents [21].

Nanoparticle effects on cellular stress pathways have been investigated in recent years [6,28,29,30]. However, to our knowledge, no prior study has specifically examined the effect of cerium dioxide nanoparticles on total rDNA copy number or on rDNA transcriptional activity in primary human cells. Since nanosized cerium dioxide and its modified products are planned for medical use, it is important to understand how ribosome biogenesis and, consequently, protein synthesis, change in response to these nanoparticles.

The aim of this study was to assess the effect of cerium dioxide nanoparticles on rDNA copy number, rDNA transcription, and total rRNA abundance (ribosome content) in cultured human skin fibroblasts.

## 2. Results

Ten HSF lines (HSF#1–10) were included in the study. Cells were cultured to sub-confluence, which was approximated visually by counting mitoses. Non-toxic amounts of cerium dioxide nanoparticles (10 µM; [10]) were added to the culture medium of sub-confluent cells for 3 and 24 h. Total rDNA copy number, the 3′ETS-rRNA transcript level (reflecting rDNA transcription at the given time), and 18S rRNA abundance (total ribosome content) were analyzed.

### 2.1. Interaction of Cerium Dioxide with Cells

**Figure 2:**
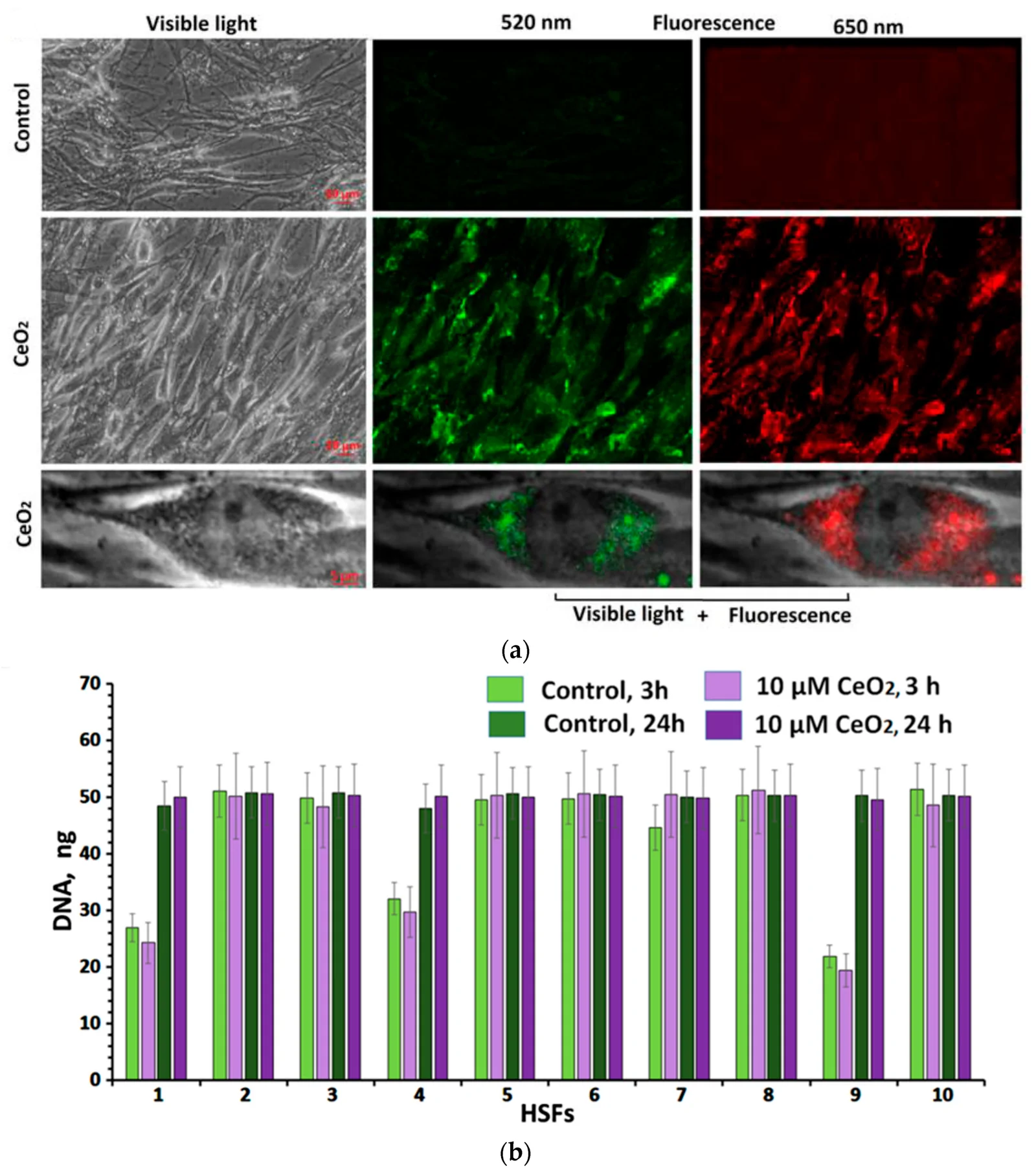
(**a**) Interaction of CeO_2_ (10 µM, 3 h) with the cells induces fluorescence of human skin fibroblasts over a wide range of wavelengths; data are shown for HSF#6 cells. (**b**) DNA mass recovered from a single well of the HSF population at the indicated time points. The DNA mass measurement is presented descriptively and is not interpreted as a direct measure of cell number, viability, proliferation, or absence of toxicity; no statistical significance markers are shown, consistent with its descriptive role. Qualitatively similar fluorescence patterns were observed across all ten donor-derived HSF lines examined.

The interaction of nanosized cerium dioxide with cells was confirmed by fluorescence microscopy (Figure 2a).

In the presence of CeO_2_, after 3 and 24 h of culture, fluorescent emission over a broad wavelength range appeared in the cell population. Fluorescence was observed on the cell surface and/or in the cytoplasm. However, no signals were detected in the nucleus or nucleolus. The fluorescence of cells in the presence of nano-CeO_2_ may be due either to fluorescence of the nanocompound itself, which catalyzes redox processes and may form fluorescent complexes/compounds with cellular components and metabolites, or to fluorescence of the cellular structures themselves (enhanced autofluorescence), for example, proteins of the NADPH oxidase family. Regardless of the origin of the fluorescence, this effect indicates interaction of the nanoparticles with the cell surface and/or penetration into the cytoplasm. The absence of detectable fluorescent signals in the nuclear region suggests that, under our experimental conditions, the nanoparticles may not enter the nucleus in substantial amounts. This observation is consistent with previous transmission electron microscopy studies showing that nanoceria predominantly localize in cytoplasmic structures and are not found in the nucleus [15,31]. However, as widefield fluorescence microscopy without nuclear counterstaining cannot definitively exclude the presence of nanoparticles in the nucleus, this interpretation remains preliminary. The observed effects on rDNA transcription and rRNA levels are therefore likely mediated by indirect mechanisms, such as modulation of cellular redox status, although direct nuclear effects cannot be completely ruled out without confirmatory studies using confocal microscopy with Z-sectioning or orthogonal methods (e.g., TEM or ICP-MS of nuclear fractions).

### 2.2. Effect of Nano-CeO_2_ on Total rDNA Copy Number in the HSF Population

To analyze the total number of rDNA copies in fibroblast genomes, we used non-radioactive quantitative hybridization (NQH) [32]. The cell lines contained from approximately 375 (HSF#4) to 490 (HSF#6) rDNA copies per diploid genome.

**Figure 3:**
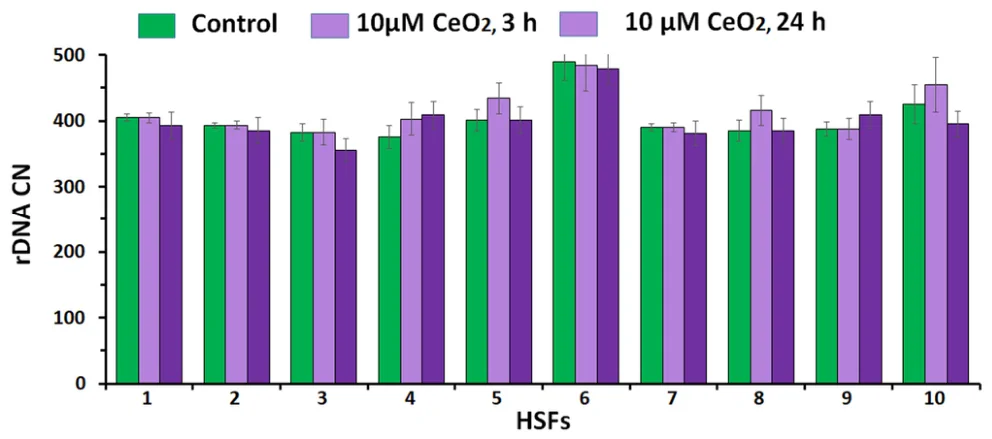
Nanosized cerium dioxide does not affect the total number of rDNA copies in the cells. The single ‘Control’ bar shown for each donor line represents the baseline rDNA copy number measured prior to nano-CeO_2_ exposure, common to both the 3 h and 24 h comparisons, since total rDNA copy number is not expected to change within this timeframe in the absence of treatment. The mean of three technical measurements and the standard error are shown.

Culturing cells for 3 or 24 h in the presence of CeO_2_ (10 µM) did not produce a change in rDNA repeat content that exceeded the reported measurement variability in DNA isolated from the fibroblast lines (Figure 3). Given the stated total experimental error of the NQH method, this result should be interpreted as no detectable change under the tested conditions rather than as evidence of equivalence or proof that smaller changes were absent. Thus, the present data support stability of the measured total rDNA copy number within the sensitivity of the method. A formal power or equivalence analysis was not pre-specified in the original study design and was not performed; given the stated total analytical error of the NQH method (11 ± 7%), changes in rDNA copy number below approximately 20% could not be reliably distinguished from measurement noise under the present design.

### 2.3. rDNA Transcription Level

**Figure 4:**
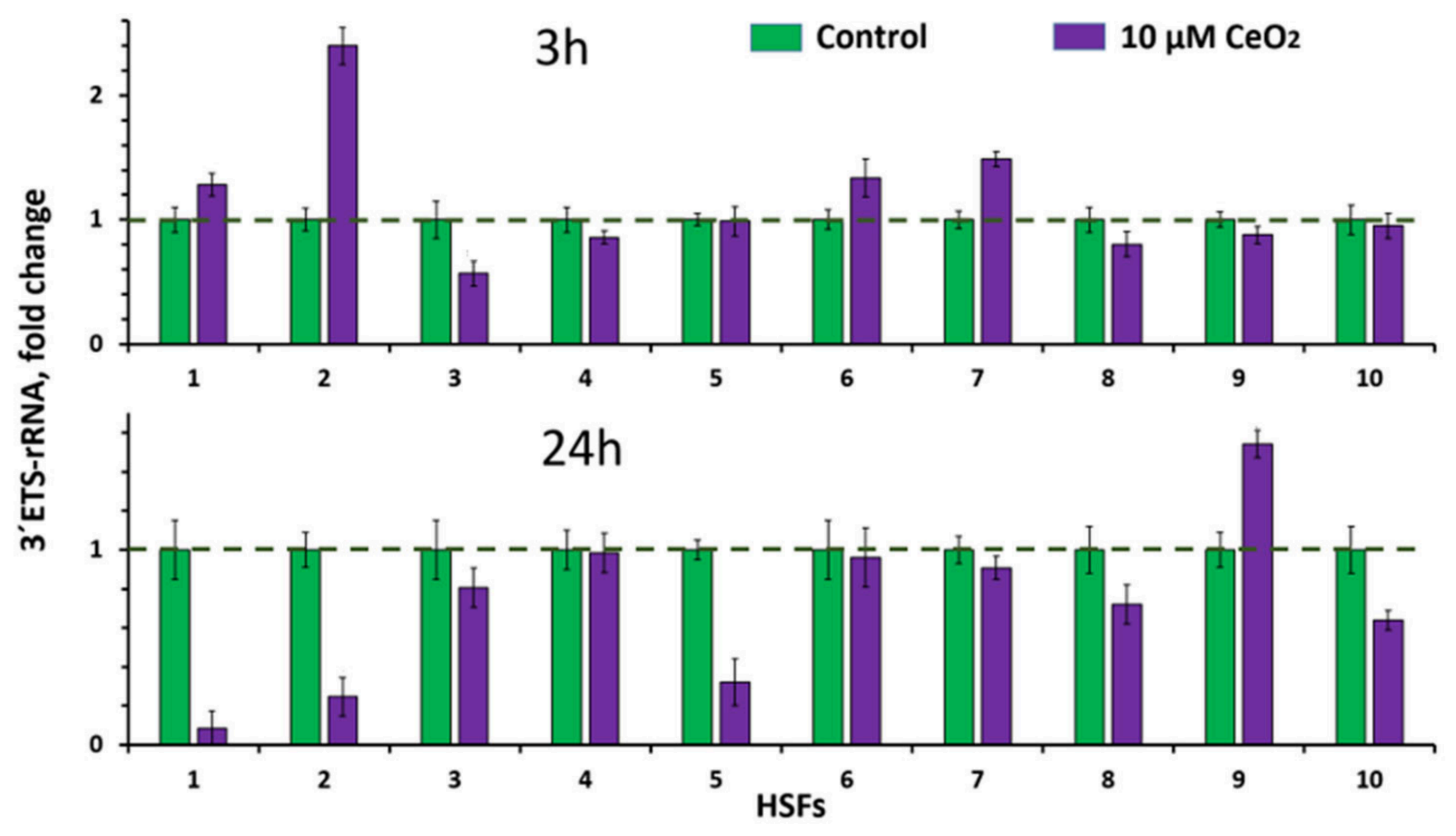
rDNA transcription level changes in the presence of cerium dioxide. The mean and the standard error are shown for each donor line. No within-line significance markers are shown, since technical-replicate comparisons at this sample size (*n* = 3 wells) cannot reach two-sided significance at α = 0.05; donor-level statistical comparisons are reported in the text and Table 1.

To assess rDNA transcription in fibroblasts, the amount of 3′ETS-rRNA was determined in RNA samples isolated from the cells. The amount of 3′ETS-rRNA reflects the actual activity of the ribosomal gene complex at the given time. The full-length 47S rRNA transcript is processed into mature rRNAs (18S, 5.8S, and 28S), whereas the remaining fragments of 47S RNA are rapidly degraded and do not accumulate in RNA over time. Figure 4 shows the 3′ETS-rRNA data obtained by real-time PCR.

The ten fibroblast lines differed substantially in rDNA transcription level. In control HSF populations after 3 h of culture, the measured rDNA transcription-related readout was positively correlated with rDNA copy number in the genome; the corresponding values obtained after incubation in the presence of nanoceria showed the same positive correlation (Table 1). Both correlations should be interpreted with caution: HSF6 (489 copies) lies outside the main cluster of donor values (374–425 copies, a gap of approximately 60 copies) and represents a high-leverage point that may disproportionately influence the strength of either fitted relationship. We therefore do not treat these correlations as a central basis for biological interpretation. Nanosized cerium dioxide did not substantially affect rDNA transcription during this period (*p* > 0.05, Wilcoxon signed-rank test). The exception was the result for HSF2: the 3′ETS-rRNA level was approximately 2.4-fold higher in the presence of nanoceria than in the corresponding control (Figure 4).

For the remaining cell lines, fluctuations in 3′ETS-rRNA relative to control did not exceed 50%. In HSF3, which had the lowest amount of rDNA in the genome, a 40% decrease in 3′ETS-rRNA was observed. Thus, short-term exposure to cerium dioxide, as a rule, does not substantially affect rDNA transcription in cultured human skin fibroblasts. In some cases, transcription activity may increase. These data indicate that the response of different cell lines to short-term nano-CeO_2_ exposure is individual.

At 24 h, cells enter a confluent state, when contact inhibition of cell division is observed. After 24 h, no positive correlation was observed between rDNA copy number and rDNA transcription level (Table 1). Nanosized cerium dioxide affects rDNA transcription during this period (*p* = 0.036, Wilcoxon signed-rank test). The presence of nanoparticles in the culture medium for 24 h was associated with lower 3′ETS-rRNA levels than in the corresponding control in lines #1, #2, #5, #8, and #10. Based on the values shown in Figure 4, the approximate decreases were 11-fold, 4-fold, 3-fold, 1.4-fold, and 1.5-fold, respectively. Only in HSF9 did the transcript amount increase relative to control in the presence of nano-CeO_2_ (approximately 1.5-fold; Figure 4). This line also showed the largest descriptive increase in DNA mass over 24 h among the ten lines (Figure 2b), which may be consistent with—but does not establish—a difference in proliferative status relative to the other lines. Thus, depending on individual characteristics, long-term culture of HSFs in the presence of nano-CeO_2_ is mainly accompanied by suppression in rDNA transcription activity.

### 2.4. Changes in 18S-rRNA Abundance in the Presence of Nanoceria

In control cells, 3 h after the start of the experiment, the measured 18S-rRNA/TBP readout was not correlated with rDNA copy number across the donor-derived lines (Table 1). The 18S-rRNA/TBP readout also showed a positive exploratory association with the 3′ETS-rRNA/TBP readout (Table 1). These correlations are presented descriptively and should not be interpreted as confirmatory evidence without donor-level analysis and correction for multiple comparisons.

**Figure 5:**
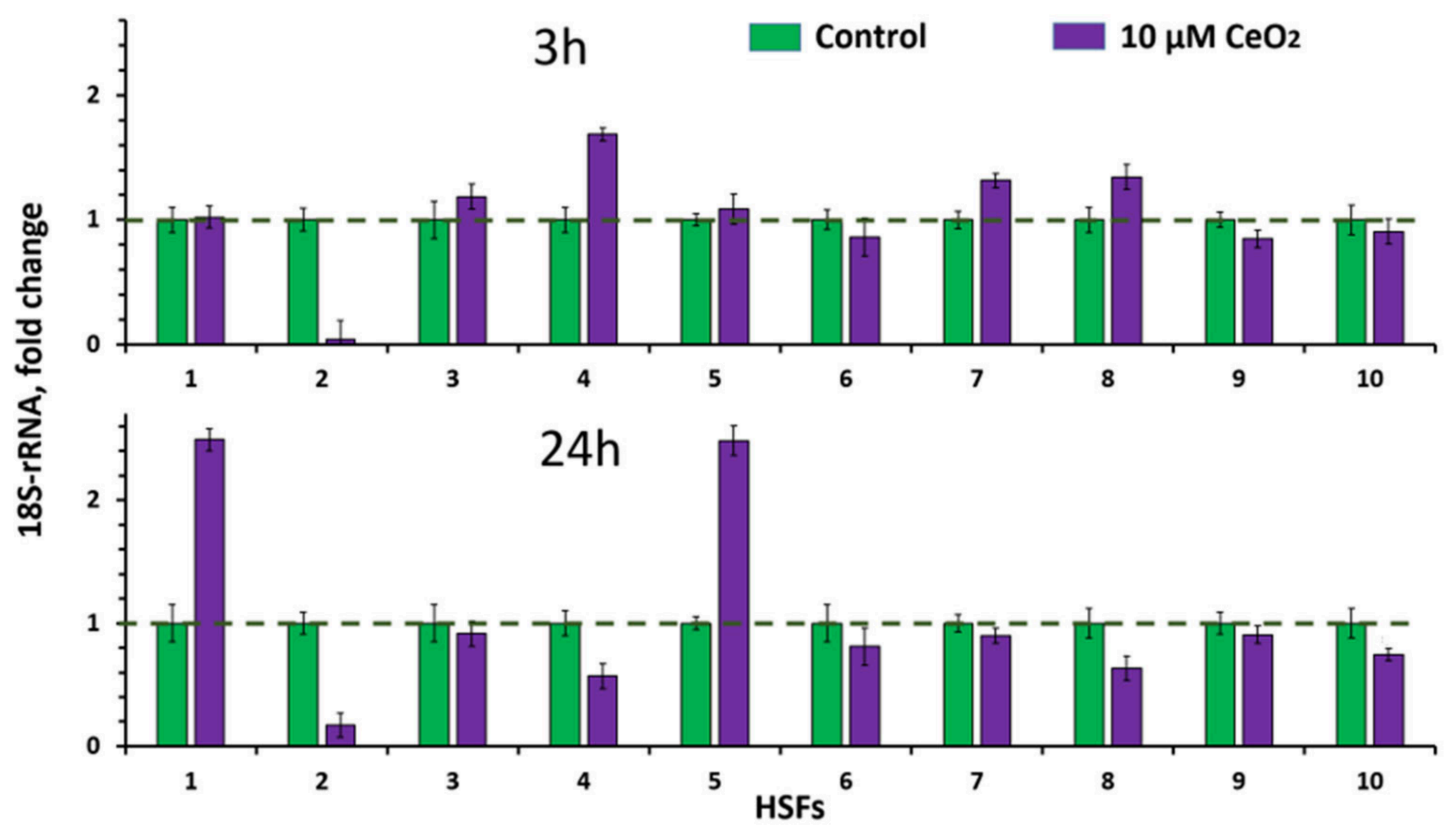
Changes in the 18S-rRNA/TBP mRNA readout in the presence of cerium dioxide. The mean (*n* = 9) and the standard error are shown for each donor line. No within-line significance markers are shown, for the same reason as in Figure 4; donor-level statistical comparisons are reported in the text and Table 1. The figure shows the same normalized readout (18S-rRNA/TBP mRNA); rDNA copy number is not included in this normalization. The mean (*n* = 9) and the standard error are shown. This readout is a relative RT-qPCR measure and should not be interpreted as an absolute measure of ribosome content (see Section 2.4).

During short-term culture of nine of the ten fibroblast lines in the presence of nano-CeO_2_, no marked changes in the 18S-rRNA/TBP readout were observed (Figure 5). The values obtained in control and nano-CeO_2_-treated cells were positively correlated across the donor-derived lines (Table 1). In HSF2, the 18S-rRNA/TBP readout was approximately 23-fold lower after exposure to nano-CeO_2_ than in the corresponding control (Figure 5). For this same line, the 3′ETS-rRNA/TBP readout was approximately 2.4-fold higher (Figure 4). These observations indicate discordant changes in the two normalized RT-qPCR readouts in HSF2; however, the present data do not establish altered 47S-rRNA processing or an increased degradation rate of ribosomal RNA. Accordingly, we describe this finding as an exposure-associated difference in the measured 18S-rRNA/TBP readout rather than as evidence for a specific post-transcriptional mechanism.

Long-term culture of cells in the absence of nano-CeO_2_ was accompanied by an increase in the 18S-rRNA/TBP readout in six fibroblast lines (#2–5, #9, and #10). Nano-CeO_2_ had little effect on this readout in most HSFs. The values obtained in control cells and cells treated with nano-CeO_2_ were positively correlated across the donor-derived lines (Table 1); this association is presented descriptively. In HSF2, the 18S-rRNA/TBP readout was approximately 5.5-fold lower after exposure to nano-CeO_2_ than in the corresponding control, whereas the readout increased in lines #1 and #5 (Figure 5). These values describe relative RT-qPCR readouts and should not be interpreted as direct measurements of total cellular rRNA or ribosome abundance.

It should be noted that interpreting changes in 18S rRNA levels requires caution. In the present study, we used TBP mRNA as a reference gene for normalization, because TBP is recognized as one of the most stable reference genes in various tissues and cell types, including fibroblasts [33]. However, we are fully aware that TBP and 18S rRNA differ by orders of magnitude in abundance and reverse-transcription efficiency, and that TBP expression may also change when cells shift from sub-confluence to confluence. Therefore, the observed sharp increases in the 18S/TBP ratio in control cells between 3 h and 24 h (e.g., in lines HSF2, HSF3, HSF4 and HSF9) may reflect both genuine rises in 18S rRNA and regulation of TBP associated with altered proliferation status. The observed differences between CeO_2_-treated and control samples are consequently interpreted as changes in the measured normalized RT-qPCR readout, not as direct evidence of altered absolute 18S-rRNA abundance, ribosome content, or a specific ribosome-biogenesis mechanism. Definitive conclusions would require independent normalization and orthogonal measurements.

## 3. Discussion

This work presents the first analysis of the response of the ribosomal gene complex in cultured cells—human skin fibroblasts from ten adult donors—to short-term (3 h) and longer-term (24 h) exposure to CeO_2_ nanoparticles added to the culture medium at a non-toxic concentration of 10 µM. Under our experimental conditions, no fluorescence signal attributable to CeO_2_ nanoparticles was detected in the nuclear/nucleolar region (Figure 2), suggesting that the particles may not enter the nucleus in substantial amounts; however, as noted above, widefield fluorescence microscopy without a nuclear counterstain cannot exclude nuclear penetration below the detection threshold of this method. Ribosomal genes were nonetheless stable under nano-CeO_2_ exposure, and the total number of rDNA copies did not change beyond the measurement uncertainty of the NQH method (Figure 3).

Despite the absence of direct contact between nanoparticles and rDNA and the stability of rDNA content in the genome, the measured rDNA-transcription-associated readout changed in the presence of nano-CeO_2_. This observation may reflect changes in the balance between transcriptionally active and inactive rDNA copies, consistent with the model of rDNA fraction distribution [19], although this study did not directly measure this balance. These changes are individual in character (Figure 4). The early response (3 h) in some lines included an increase in the 3′ETS-rRNA readout, consistent with—but not direct evidence of—a shift toward actively transcribed (fraction 1) rDNA copies. However, longer exposure to the nanoparticles is accompanied by a relative decrease in this readout in most cell lines.

Based on these indirect readouts, we propose the following hypothesis: CeO_2_ nanoparticles modulate ribosome biogenesis by shifting the balance between transcriptionally active (fraction 1) and poised (fraction 2) rDNA copies, without altering the total number of rDNA repeats. This hypothesis is motivated by the observed changes in 3′ETS-rRNA (a marker of ongoing transcription) and 18S rRNA levels, coupled with the stability of total rDNA copy number. We emphasize that this interpretation remains indirect, and direct testing of this hypothesis would require fractionation experiments assessing the methylation status and nucleosome occupancy of individual rDNA copies [17,18]. Such studies are planned for future investigations.

The level of 3′ETS-rRNA is a well-established marker of ongoing rDNA transcription, as its short half-life reflects recent transcriptional events [34]. Moreover, changes in 18S-rRNA abundance without alterations in total rDNA copy number have been previously linked to shifts in the methylation status of rDNA repeats, which are known to modulate the proportion of active versus silent copies [35,36]. Thus, our data are in line with the concept that rDNA functional fractions can be rearranged without changing total copy number.

It is known that the ribosomal gene complex responds to many cellular processes, including proliferative stimuli, stress, and changes in reactive oxygen species, by increasing transcriptional activity [17,18]. The DNA-mass measurements (Figure 2b) did not show a clear treatment-related pattern across the donor panel, but as this measurement is descriptive and was not designed to assess proliferation directly, we do not draw a proliferation-related conclusion from it; the MTT assay (Figure S2) addresses viability, not proliferation rate.

The observed changes in rDNA transcription activity may be related to changes in the intensity of redox processes in cells. It is well established that nanoceria can modulate intracellular reactive oxygen species (ROS) levels through its antioxidant and redox cycling properties [37]. Based on this, we hypothesize that the reduced rDNA transcription activity observed in the presence of nano-CeO_2_ may reflect a decrease in cellular stress level, potentially mediated by ROS scavenging. This interpretation is consistent with the known ability of CeO_2_ nanoparticles to lower ROS levels in various cell types [37]. However, we acknowledge that direct measurements of intracellular ROS were not performed in the present study. Therefore, this mechanistic explanation remains a hypothesis that requires direct experimental confirmation in future investigations, for example using ROS-sensitive fluorescent probes.

The potential ability of CeO_2_ nanoparticles to reduce nucleolar activity in non-dividing cells without disrupting the integrity of the ribosomal gene complex requires further study. It has previously been shown that increased lifespan achieved by various methods, including caloric restriction, leads to a reduction in the transcription of both rRNA and ribosomal protein genes. Slowing ribosome biogenesis and protein biosynthesis is considered a way to increase lifespan, including in manifestations of accelerated aging [38,39,40]. Non-toxic CeO_2_ nanoparticles have potential as therapeutic agents that reduce abnormal nucleolar activity caused by various factors [29]. A decrease in rDNA transcription, together with the antioxidant properties of CeO_2_ nanoparticles, may reduce chronic inflammation (inflammaging), which contributes to age-associated diseases and reduced lifespan. Integrative studies of the gene targets are perspective to develop nanoparticle-based drugs [29].

Our findings that CeO_2_ nanoparticles modulate rDNA transcription and rRNA levels without altering total rDNA copy number are consistent with the emerging concept of nucleolar stress as a cellular response to various insults [41,42]. Recent reviews have highlighted that the nucleolus functions not only as the site of ribosome biogenesis but also as a critical stress sensor and signaling hub, responding to nutrient starvation, DNA damage, and oxidative stress [41,43,44]. Nucleolar stress frequently leads to dynamic reorganization of nucleolar proteins and changes in rDNA transcription [42]. Moreover, disruption of ribosome biogenesis regulation can result in nucleolar stress, activating p53-dependent and p53-independent checkpoint pathways leading to cell cycle arrest or apoptosis [43]. The ability of CeO_2_ nanoparticles to reduce rDNA transcription activity in confluent fibroblasts may reflect a stress-relieving effect, potentially mediated by their antioxidant properties, rather than a direct toxic insult. This interpretation aligns with recent work showing that agents that regulate nucleolar activity may have therapeutic potential in conditions associated with abnormal protein accumulation and oxidative stress [41]. Furthermore, the donor-specific responses observed in our study underscore the importance of interindividual variability in nucleolar stress responses, an aspect that warrants further investigation in the context of nanomedicine and personalized approaches to geroprotection.

Limitations. The interpretation of altered rDNA fraction balance and nucleolar reorganization reflects a model consistent with the transcriptional readouts rather than direct measurement, since no methylation-sensitive fractionation assay, chromatin-based method (psoralen crosslinking, ChIP for UBF/Pol I), or quantitative nucleolar imaging (immunofluorescence for fibrillarin, nucleolin, or UBF) was performed. The absence of detectable nuclear fluorescence likewise does not exclude nuclear entry of nanoparticles below the resolution of widefield microscopy without nuclear counterstaining. The proposed redox-mediated mechanism is a literature-based hypothesis, as intracellular ROS was not measured directly. In addition, the 18S-rRNA readout was normalized only to TBP mRNA; no independent, non-transcriptional normalizer (e.g., total RNA input, DNA mass per well, or cell number) was applied at matching resolution. Although DNA mass was recorded descriptively for a single well per condition (Figure 2b), this measurement does not have the well-level correspondence needed to renormalize the 18S-rRNA/TBP readout, which is averaged across three wells (*n* = 9 technical measurements). Consequently, the present data cannot determine whether the large HSF2 difference reflects a genuine change in 18S-rRNA or co-varying regulation of the TBP denominator, although the contribution of the latter is likely small. Finally, the statistical analysis remains descriptive at the donor level; a pre-registered paired analysis with multiplicity correction and a formal power/equivalence assessment was beyond the scope of this dataset and is identified as a priority for future work.

## 4. Materials and Methods

### 4.1. Synthesis and Characterization of Nanoparticles

Nanosized CeO_2_ was synthesized by thermal hydrolysis of ammonium hexanitratocerate(IV) (#215473, Sigma, St. Louis, MO, USA) [45]. The aqueous solution of the starting compound was kept at 95 °C for 24 h. The resulting precipitate was washed at least three times with isopropanol and redispersed in deionized water. Residual isopropanol was removed by boiling.

Powder X-ray diffraction analysis of the dried CeO_2_ sol was performed on a DX-2700BH (Haoyuan, China) diffractometer, using CuKα-radiation (λ = 1.5406 Å) in the 2θ range of 10−60° with 0.02° 2θ steps and a signal accumulation time of 2.0 s per point. Scanning transmission electron microscopy (STEM) imaging was obtained at 30 kV on an Amber GMH (Tescan, Brno, Czech Republic) scanning electron microscope equipped with a R-STEM detector (Tescan, Brno, Czech Republic). The ImageJ software (Version 1.54k) was used for the particle size analysis. Absorption spectra of CeO_2_ sols were recorded at room temperature on a Cary 4000 UV-Vis spectrophotometer (Agilent Technologies, Santa Clara, CA, USA) in quartz cuvettes with an optical path length of 1.0 cm. The hydrodynamic diameter of the nanoparticles was measured with a Photocor Complex analyzer (radiation power 25 mW, diode laser, λ = 650 nm) (Photocor, Moscow, Russia). Measurements were performed at room temperature at a scattering angle of 90°. The ζ-potential was measured with a Nano ZS Zetasizer (Malvern Panalytical, Worcestershire, UK) according to ISO/TR 19997:2018. Fluorescence spectra (λ_ex_ = 250 nm, slit width 5 nm) were recorded on a FluoroLog 3 spectrofluorimeter (HORIBA Jobin Yvon, Kyoto, Japan) [46].

The concentration of the electrostatically stabilized CeO_2_ sol, determined by thermogravimetric analysis, was 0.120 ± 0.01 mol/L (21 g/L). Powder X-ray diffraction analysis of the dried CeO_2_ sol (Figure S1a) confirmed single-phase cerium dioxide with a fluorite structure (PDF2 34-0394). The CeO_2_ crystallite size, calculated using the Scherrer equation, was 3.0 ± 0.4 nm. Dynamic light scattering gave a mean hydrodynamic diameter of 12 ± 2 nm, indicating moderate aggregation of particles in water. The polydispersity index (PDI) was 0.006, consistent with a narrow size distribution. STEM imaging (Figure S1b) showed that the CeO_2_ nanoparticles formed aggregates ranging from 11 to 16 nm upon drying. The UV–vis absorption spectrum of the CeO_2_ sol (Figure S1c) displayed a characteristic absorption band at 280–300 nm, typical of cerium dioxide and corresponding to a band gap of ~3.3 eV. The zeta potential of the sol was +39.3 ± 0.4 mV at pH ~4.0, indicating high colloidal stability. The fluorescence spectrum of the CeO_2_ sol exhibited an intense emission maximum at 355 nm (Figure S1d).

Accurate interpretation of the biological effects of CeO_2_ nanoparticles requires careful consideration of protein adsorption in culture media containing 10% FBS. Bovine serum albumin, the predominant protein in FBS, shares 98% structural and functional homology with human serum albumin (HSA) [47]. Studies on CeO_2_–HSA conjugates demonstrate that albumin interaction preserves the crystalline structure and primary size of the nanoparticles [48,49]. At the same time, it increases their hydrodynamic diameter and reduces their surface charge. These changes are consistent with protein corona formation [49]. Critically, these modifications do not compromise colloidal stability. CeO_2_ sols remain stable and do not form large insoluble aggregates under the reported conditions [48,49].

### 4.2. Cell Culture

Human skin fibroblast (HSF) cultures from ten donors (aged 18 to 55 years; 7 men and 3 women) (passage 4) were obtained from the collection of the Research Centre for Medical Genetics named after Academician N.P. Bochkov. Cells were cultured in Dulbecco’s Modified Eagle Medium supplemented with 10% fetal bovine serum (HyClone Laboratories, Logan, UT, USA), 2 mM glutamine, 50 U/mL penicillin, and 50 µg/mL streptomycin at 37 °C in a 5% CO_2_ incubator. A culture with fewer than 10% of cells in S phase was considered sub-confluent. Each HSF cell line was cultured in three wells of a six-well plate. To analyze the cellular response, CeO_2_ nanoparticles were added to the culture medium at a final concentration of 10 µM (equivalent to 1.72 µg/mL in terms of CeO_2_) for 3 h and 24 h. This concentration was chosen based on our previous studies demonstrating that nanoceria is non-toxic up to millimolar concentrations in primary human fibroblasts [10,50]. To directly confirm the absence of cytotoxicity in the specific cell model used in the present study, we performed an MTT viability assay in HSF cultures across a concentration range of 0.012 μM to 7 mM after 24 h of incubation (Figure S2); no significant decrease in viability was observed up to 1.6 mM, confirming that 10 μM is well within the non-toxic range for this model. Each HSF sample was analyzed in triplicate (a total of nine samples were analyzed: three wells, with three analyses performed for each well).

The study was conducted in accordance with the Declaration of Helsinki and approved by the Ethics Committee of the Research Centre for Medical Genetics (Protocol No. 4 of 15 March 2019). All participants signed informed written consent after the procedures had been fully explained to them.

### 4.3. Real-Time Polymerase Chain Reaction

RNA was isolated from cells using YellowSolve kits (Clonogen, St. Petersburg, Russia) or Trizol reagent (Invitrogen, Carlsbad, CA, USA) according to the manufacturer’s protocol (https://documents.thermofisher.com/TFS-Assets%2FLSG%2Fmanuals%2Ftrizol_reagent.pdf, accessed on 22 March 2021). Phenol-chloroform extraction and precipitation were performed using a chloroform/isopropyl alcohol mixture (49:1). RNA concentration was determined using the Quant-iT RiboGreen RNA reagent (R11491, Invitrogen, Carlsbad, CA, USA) on an EnSpire plate reader (PerkinElmer, Waltham, MA, USA) at excitation and emission wavelengths of 487 and 524 nm, respectively. PCR was carried out with specific primers and SYBR Green dye on a StepOnePlus instrument (Applied Biosystems, Foster City, CA, USA). Primer design and synthesis were performed by Eurogen (Eurogen, Moscow, Russia).

Reverse transcription was performed using random hexamer + oligo-dT primers according to the manufacturer’s protocol (Sileks, Moscow, Russia). RNA samples were treated with DNase prior to reverse transcription to eliminate genomic DNA contamination. Primer sequences, amplicon lengths, amplification efficiencies, and specificity evidence (melt-curve analysis) for the 3′ETS-rRNA, 18S-rRNA, and TBP assays are provided in Supplementary Table S1.

The 25-µL PCR reaction mixture contained 2.5 µL of PCR buffer (700 mmol/L Tris-HCl, pH 8.6; 166 mmol/L ammonium sulfate; 35 mmol/L MgCl2), 2 µL of dNTP solution at a concentration of 1.5 mmol/L, 1 µL of primer solution at a concentration of 30 pmol/L, and cDNA. PCR conditions were optimized individually for each primer pair. After preliminary denaturation for 4 min at 95 °C, 40 amplification cycles were performed as follows: 94 °C for 20 s, 56–62 °C for 30 s, and 72 °C for 30 s, followed by a final elongation step at 72 °C for 5 min. Data were processed using a calibration curve; measurement error was 2%.

### 4.4. Non-Radioactive Quantitative Hybridization (NQH)

DNA was isolated using a standard method described previously [32]. Briefly, 2 mL of a solution containing 2% sodium lauroyl sarcosinate, 0.04 M EDTA, and 150 µg/mL RNase A (Sigma, St. Louis, MO, USA) was added to the cell mass and incubated for 45 min at 37 °C, after which proteinase K (200 µg/mL, Promega, Madison, WI, USA) was added for 24 h at 37 °C. The lysates were sequentially extracted with an equal volume of phenol, then phenol/chloroform/isoamyl alcohol (25:24:1), and finally chloroform/isoamyl alcohol (24:1). To precipitate DNA, 1/10 volume of 3 M sodium acetate (pH 5.2) and 2.5 volumes of cold ethanol were added. DNA was collected by centrifugation (10,000× *g*, 15 min, 4 °C), washed with 70% ethanol, and dissolved in water. DNA concentration was determined in two stages as described previously [32]. DNA was quantified fluorimetrically using PicoGreen reagent for double-stranded DNA quantification (Invitrogen, Carlsbad, CA, USA). DNA concentration in the sample was calculated from a calibration curve built using a DNA standard. Measurements were performed on an EnSpire instrument (PerkinElmer, Waltham, MA, USA) at excitation and emission wavelengths of 488 and 528 nm.

The NQH method is based on complementary hybridization of DNA immobilized on a filter with a biotinylated DNA probe. NQH was used to quantify the number of rDNA repeats [32]. Briefly, five standard DNA samples with known repeat content were applied to the filter. After hybridization and biotin detection using a streptavidin–alkaline phosphatase conjugate (with BCIP and NBT substrates), the filter was scanned and signal intensities in spots were measured with specialized software. A calibration curve was built to reflect the relationship between signal intensity and repeat copy number in the genome. The relative standard error of the NQH method did not exceed 5 ± 2%. The main contribution to the total experimental error came from the DNA extraction step. The total standard error was 11 ± 7%.

The DNA probe used for rDNA quantification contained rDNA sequences (5836 bp) cloned into the EcoRI site of vector pBR322. The rDNA fragment covered positions −515 to 5321 of human rDNA (GenBank No. U13369). Each sample was analyzed in technical triplicate; the mean value was used for subsequent donor-level analysis.

### 4.5. Fluorescence Microscopy

CeO_2_ solution was added to the culture medium for 3 h or 24 h (10 µM). Cells were then washed three times with phosphate-buffered saline (PBS). Fluorescence images were obtained using an Axio Scope.A1 microscope (Carl Zeiss, Oberkochen, Germany). Image acquisition was performed using a 63× objective (NA 1.25) on the Axio Scope.A1 microscope. Two emission channels were acquired at approximately 520 nm and 650 nm, consistent with standard GFP-type (excitation ~470 nm) and TexasRed-type (excitation ~560 nm) filter sets, respectively, with an exposure time of 500 ms for each channel. At least three independent fields of view were acquired per sample. The exact filter cube designation was not recorded at the time of acquisition.

### 4.6. Statistical Analysis

Each donor-derived HSF line was treated as an independent biological unit (*n* = 10). For each donor and condition, cells were cultured in three independently prepared wells, and each well was measured in technical triplicate. Technical replicates were averaged within each well; the three well-level values were then averaged to obtain one donor-level value per condition for inferential analysis. This averaging procedure was applied separately for each method: for RT-qPCR (Section 2.3), the three well-level triplicate means were averaged per donor per condition; for the NQH assay (Section 2.4), the three technical replicate measurements obtained from each donor-derived DNA sample were averaged directly to yield one donor-level value, without the intermediate well-replication step used for RT-qPCR.

The significance of observed differences was assessed using the non-parametric Mann–Whitney U test. Values of *p* < 0.05 were considered statistically significant. The effects of CeO_2_ on the HSF cell lines were evaluated using the Wilcoxon signed-rank test. Correlation analysis was carried out using nonparametric test (Spearman’s coefficient). The Benjamini-Hochberg method (adj.p) was used to correct for multiple comparisons. Results with *p* ≤ 0.05 after correction were considered statistically significant. Data were processed using Excel and Microsoft Office (Microsoft, Redmond, WA, USA), StatPlus2007 Professional, and StatGraphics (Statgraphics Technologies, The Plains, VA, USA).

## 5. Conclusions

Cerium dioxide nanoparticles altered rDNA-transcription-associated in primary human skin fibroblasts in a donor- and time-dependent manner, without a detectable change in total rDNA copy number. These findings are consistent with, but do not directly demonstrate, modulation of ribosome biogenesis or a shift in the balance between transcriptionally active and inactive rDNA copies; testing either hypothesis directly would require orthogonal measurements (e.g., independent normalization of rRNA abundance, methylation- and nucleosome-based rDNA fractionation) beyond the scope of the present study. These results indicate that, when assessing the safety and therapeutic potential of nano-CeO_2_, its effects on rDNA transcriptional activity should be considered alongside its known antioxidant and redox properties and warrant further mechanistic investigation.

## Figures and Tables

**Table 1:** Exploratory correlation matrix for the measured parameters. The correlation coefficient (r) and the adjusted nominal *p*-value are shown for each pairwise comparison. Grey shading and bold correlation coefficients indicate statistically significant correlations (adjusted *p* < 0.05).

	rDNA CN	3′ETS, Control (3 h)	3′ETS, CeO_2_(3 h)	3′ETS, Control (24 h)	3′ETS, CeO_2_ (24 h)	18S, Control (3 h)	18S, CeO_2_ (3 h)	18S, Control (24 h)	18S, CeO_2_ (24 h)
**rDNA CN**	—	**0.71**	**0.73**	0.01	0.11	0.51	0.42	–0.16	–0.22
	—	**0.03**	**0.02**	0.97	0.75	0.12	0.22	0.66	0.54
3′ETS, control (3 h)	**0.71**	—	**0.81**	0.11	0.21	0.45	0.50	–0.13	–0.34
	**0.03**	—	**0.01**	0.77	0.56	0.16	0.14	0.72	0.33
**3′** **ETS, CeO_2_ (3 h)**	**0.73**	**0.81**	—	0.27	0.08	0.64	0.46	0.22	–0.05
	**0.02**	**0.01**	—	0.45	0.82	0.05	0.18	0.54	0.89
3′ETS, control (24 h)	0.01	0.11	0.27	—	0.40	–0.02	–0.26	0.60	0.20
	0.97	0.77	0.45	—	0.25	0.95	0.47	0.07	0.57
**3′** **ETS, CeO_2_ (24 h)**	0.11	0.21	0.08	0.40	—	0.08	0.08	–0.18	–0.41
	0.75	0.56	0.82	0.25	—	0.82	0.84	0.63	0.24
18S, control (3 h)	0.51	0.45	0.64	–0.02	0.08	—	**0.81**	–0.10	–0.38
	0.12	0.16	0.05	0.95	0.82	—	**0.01**	0.78	0.27
**18S, CeO_2_ (3 h)**	0.42	0.50	0.46	–0.26	0.08	**0.81**	—	–0.38	–0.57
	0.22	0.14	0.18	0.47	0.84	**0.01**	—	0.28	0.09
18S, control (24 h)	–0.16	–0.13	0.22	0.60	–0.18	–0.10	–0.38	—	**0.71**
	0.66	0.72	0.54	0.07	0.63	0.78	0.28	—	**0.03**
**18S, CeO_2_** **(24 h)**	–0.22	–0.34	–0.05	0.20	–0.41	–0.38	–0.57	**0.71**	—
	0.54	0.33	0.89	0.57	0.24	0.27	0.09	**0.03**	—

## Data Availability

The original contributions presented in this study are included in the article/Supplementary Materials. Further inquiries can be directed to the corresponding author.

## Supplementary Materials

The following supporting information can be downloaded at: https://www.mdpi.com/article/10.3390/ijms27198913/s1.

## Abbreviations

The following abbreviations are used in this manuscript:

BCIP	5-bromo-4-chloro-3-indolyl phosphate
DME(M)	Dulbecco’s Modified Eagle Medium
dNTPs	deoxynucleoside triphosphates
EDTA	ethylenediaminetetraacetic acid
3′ETS	external transcribed spacer
FBS	fetal bovine serum
NBT	Nitro Blue Tetrazolium
NQH	non-radioactive quantitative hybridization
PBS	phosphate-buffered saline
PCR	polymerase chain reaction
rDNA	ribosomal DNA
rRNA	ribosomal RNA
