# Supplementary material for: Cerium Dioxide Nanoparticles Modulate Ribosomal DNA Transcriptional Activity in Cultured Human Skin Fibroblasts
Source: Int J Mol Sci. 2026 Oct 7;27(19):8913. doi: 10.3390/ijms27198913 (PMC13665379; doi:10.3390/ijms27198913)
Supplement: Supplementary file 1 [file ijms-27-08913-s001.zip › ijms-4519813-supplementary.pdf]

Supplementary Materials

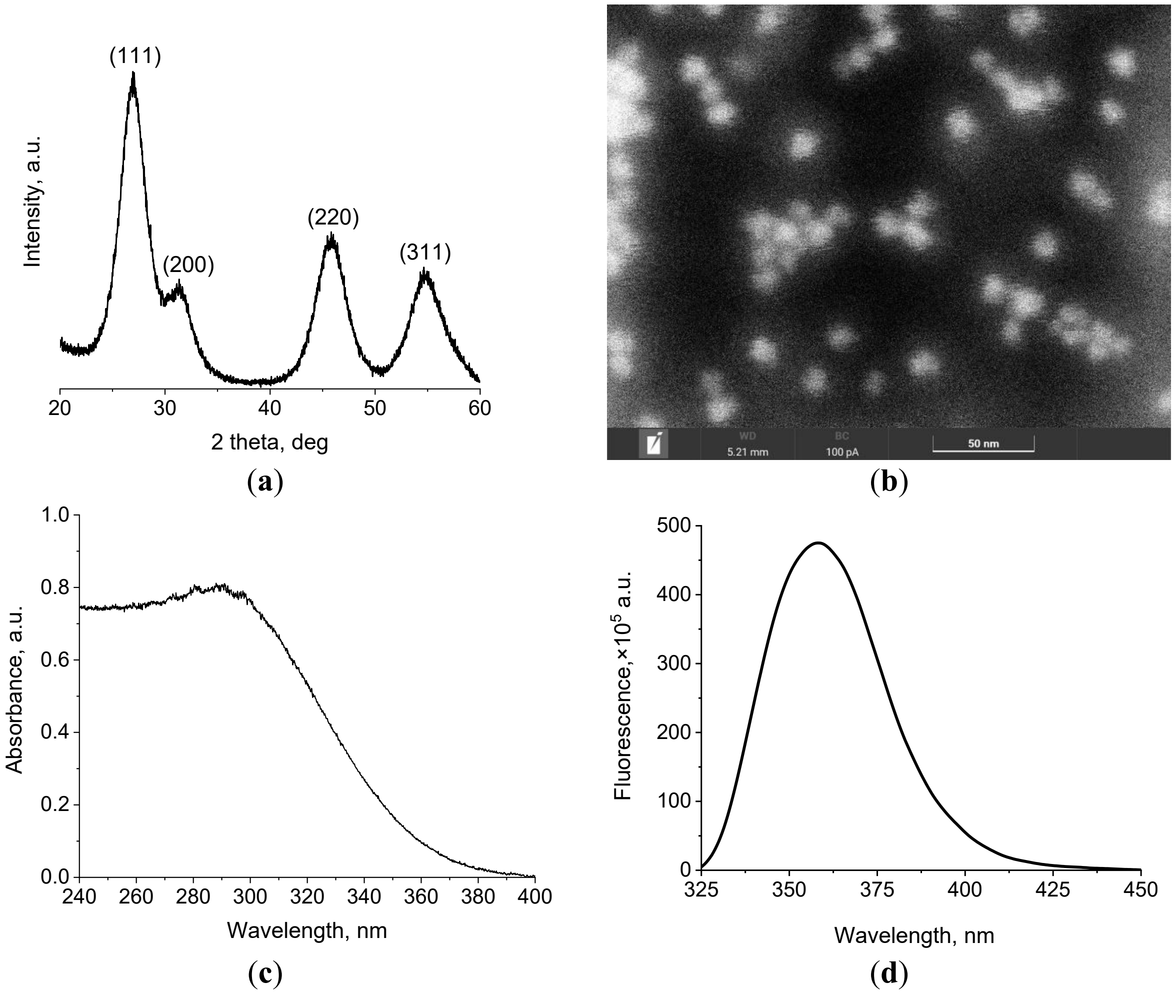

Figure S1. Physicochemical characteristics of the ceria sol: (**a**) – XRD pattern of the dried sol; (**b**) – STEM image; (**c**) – UV-vis absorption spectrum and (**d**) – fluorescence spectrum (λ_{ex} = 250 nm).

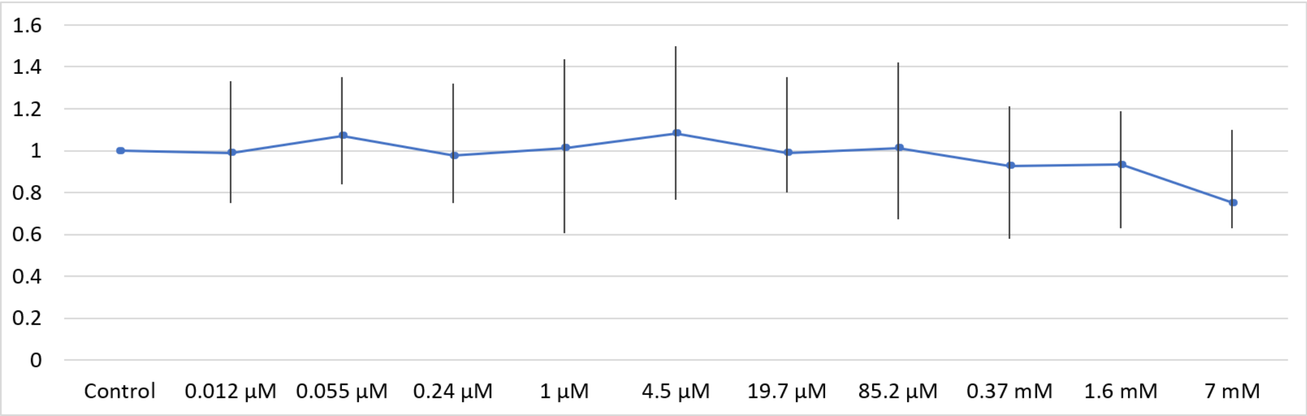

Figure S2. MTT assay of HSF viability after 24 h incubation with CeO_2 nanoparticles across a wide concentration range (0.012 μM – 7 mM). Values are expressed relative to untreated control (mean ± SD). Cell viability remained unaffected at concentrations up to 1.6 mM, confirming that the 10 μM concentration used in the main experiments was non-toxic to this cell model after 24 h.

Table S1. Primer sequences, amplicon lengths, amplification efficiencies, and specificity evidence for the RT-qPCR assays used in this study

Target	Primer sequence (F/R)	Amplicon length (bp)	Efficiency (%)	Specificity evidence
3′ETS-rRNA	F: 5'- CCCGCTTCTTCGGTTCC -3'; R: 5'- GGCTCCCAAACCACGCT -3'	78	95%	Single melt peak (SYBR Green)
18S-rRNA	F: 5'- GCCCGAAGCGTTTACTTTG -3'; R: 5'- CCGCGGTCCTATTCCATTAT -3'	93	96%	Single melt peak (SYBR Green)
TBP	F: 5'-GCCCGAAACGCCGAATAT-3'; R: 5'-CCGTGGTTCGTGGCTCTCT-3'	73	98%	Single melt peak (SYBR Green)
